# Posttreatment Non-Improved Vocal Cord Mobility Indicates the Need of Salvage Surgery for Hypopharyngeal Carcinomas

**DOI:** 10.3389/fonc.2020.600599

**Published:** 2021-01-08

**Authors:** Yu-qin He, Xi-wei Zhang, Yi-ming Zhu, Xiao-guang Ni, Ze-hao Huang, Chang-ming An, Jun-lin Yi, Shao-yan Liu

**Affiliations:** ^1^ Department of Head and Neck Surgery, National Cancer Center/National Clinical Research Center for Cancer/Cancer Hospital, Chinese Academy of Medical Sciences, Peking Union Medical College, Beijing, China; ^2^ Department of Endoscopy, National Cancer Center/National Clinical Research Center for Cancer/Cancer Hospital, Chinese Academy of Medical Sciences, Peking Union Medical College, Beijing, China; ^3^ Department of Radiation Oncology, National Cancer Center/National Clinical Research Center for Cancer/Cancer Hospital, Chinese Academy of Medical Sciences, Peking Union Medical College, Beijing, China

**Keywords:** salvage therapy, radiotherapy, hypopharyngeal neoplasms, prognosis, vocal fold paralysis

## Abstract

**Introduction:**

We aimed to analyze the relationship between the changed status of vocal cord mobility and survival outcomes.

**Methods:**

Seventy-eight patients with dysfunctional vocal cords and hypopharyngeal carcinomas accepted non-surgical treatment as the initial therapy between May 2009 and December 2016. Vocal cord mobility was assessed before and after the initial non-surgical treatment. The cord mobility status was classified as normal, impaired, and fixed. Patients with improved mobility (IM) (n =56) were retrospectively analyzed for disease-free survival (DFS), recurrence-free survival (RFS), and overall survival (OS) and compared with 22 patients with non-improved mobility (non-IM).

**Results:**

Fifty-six (71.8%) patients had improved cord mobility after the initial non-surgical treatment. The non-improved cord mobility was significantly associated with shortened DFS (*P=*0.005), RFS (*P=*0.002), and OS (*P*<0.001). If non-improved cord mobility was regarded as an indicator for local-regional recurrence within 1 year, the sensitivity and the specificity were 60.9%, 87.5% respectively. The multivariate analysis showed that improved cord mobility (*P=*0.006) and salvage surgery (*P=*0.015) were both independent protective factors for OS.

**Conclusion:**

Changes in cord mobility are a key marker for predicting prognosis. Non-improved cord mobility may indicate a high possibility of a residual tumor, therefore, patients whose cord mobility remains dysfunctional or worsens after non-surgical treatment might need an aggressive salvage strategy.

## Introduction

Although the prognosis of hypopharyngeal squamous cell carcinoma (HPSCC) has improved in the last decade, HPSCC is still associated with the worst survival outcomes among all head and neck cancers. The 5-year survival rate of patients with advanced disease (stages III–IV) ranges from 30 to 54% (1, 2). Pretreatment vocal cord fixation is a significantly poor prognostic factor in HPSCC. According to the 8^th^ American Joint Committee Cancer (AJCC) staging atlas (3), hemilarynx fixation is one of the indicators used to upgrade T1–2 to T3–4 regardless of the size of the primary tumor. The mobility of the vocal cord might affect the choice of conservative surgery.

Although advanced HPSCC has no standard treatment strategy, it is no doubt that advanced HPSCC should be treated with combined therapy by a multidisciplinary team. Traditional open surgery is always followed by unacceptable functional loss. Therefore, the treatment strategy is gradually shifting from surgery-based treatment to radiotherapy-based treatment for organ preservation. However, salvage surgery still plays an important role in the treatment of advanced HPSCC and relapse, but the proper time for surgical invention and how to select candidates who would benefit from the sacrifice of function for survival purposes remain questionable.

In clinical practice, we have observed that the cord mobility status might change after non-surgical treatment in patients with laryngeal and hypopharyngeal cancers. A few studies have analyzed this phenomenon in patients with laryngeal cancers, but the authors did not agree on whether remobility of the vocal cord could predict a good prognosis (3, 4). No literature has focused on the value of improved cord mobility after non-surgical therapy in HPSCC. It is unclear whether improved cord mobility could be a prognostic signal or a predictive factor of therapeutic efficacy. In other words, we wonder whether non-improved cord mobility indicates a residual tumor after non-surgical therapy and the need for salvage surgery. To the best of our knowledge, this is the first study to evaluate the prognostic value of a changed vocal cord mobility status after non-surgical therapy in HPSCC. We also evaluated the role of salvage surgery in survival outcomes.

## Materials and Methods

### Patients

This retrospective study focused on patients with dysfunctional vocal cords and HPSCC. All patients accepted non-surgical treatment as the initial therapy at the National Cancer Center (China) between May 2009 and December 2016. The study was approved by the institutional review board of the National Cancer Hospital of the Chinese Academy of Medical Sciences, Beijing, China. All patients wrote informed consent before treatment. Each therapeutic strategy was discussed by a multidisciplinary team before treatment. Non-surgical treatment included a radiotherapy-based strategy with or without systemic therapy. Systemic therapy included concurrent or neoadjuvant. Patients who could not tolerate a total dose of 66–70 Gy and who had distant metastasis at the initial diagnosis were excluded. There were no toxicity-mandated breaks or delays during radiotherapy. Salvage surgery included at least one of the following: neck dissection, partial pharyngectomy, and total pharyngolaryngectomy. Tumor (T) classification and lymph node (N) classification were defined through imaging examinations and fiberoptic laryngoscopy according to the criteria of the AJCC 7^th^ edition staging system (5).

### Vocal Cord Mobility

We reviewed the results of fiberoptic laryngoscopy in all enrolled patients. All classifications of the status of vocal cord mobility were carried out by one experienced endoscopist (who performed fiberoptic laryngoscopy over 3 years). Vocal cord mobility was assessed at least twice, including before and within 3 months after the initial non-surgical treatment. The cord mobility status was classified as normal or dysfunctional mobility. Dysfunctional cord mobility included impaired and fixed cord mobility. The impaired cord mobility was defined as the mobility of vocal cord weaken than that of the healthy side. Improved mobility (IM) consisted of complete response (CR) and partial response (PR). CR was defined as cord mobility that changed from impaired/fixed to normal after non-surgical treatment, and PR was defined as cord mobility that changed from fixed to impaired mobile after non-surgical treatment. If the mobility status of the patient remained fixed/impaired after non-surgical treatment, it was defined as stable dysfunction (SD). If the mobility status of the patient changed from impaired to fixed after non-surgical treatment, it was defined as progressive dysfunction (PD). PD and SD were both regarded as non-improved mobility (non-IM).

### Survival Outcomes

Outcomes consisted of recurrence-free survival (RFS), disease-free survival (DFS), and overall survival (OS). OS was calculated from the date of the completion of initial non-surgical treatment and was censored at the date of all-cause death or the last follow-up. Local-regional recurrence, new distant metastasis, and persistent disease were censored as recurrence. New secondary primary cancers and recurrence were end events of DFS and were diagnosed by an imaging examination or biopsy. If patients did not accept salvage surgery, RFS and DFS were calculated from the date of the completion of initial non-surgical treatment; If patients accepted salvage surgery, RFS and DFS were calculated from the date of salvage surgery. Imaging examinations have done on all individuals every 3 months for the first 2 years, followed by every 6 months for the next 3 years, and annually thereafter. Based on the results, salvage surgeries have performed to the patients who were confirmed stable disease, incomplete response or relapse after radiotherapy. All salvage surgery should be performed within 6 months after non-surgical treatment. Salvage surgery beyond 6 months and with positive pathological results was regarded as local-regional recurrence.

### Statistical Analysis

All categorical variables were estimated using two-sided Fisher’s exact tests, and all continuous variables were estimated using the Wilcoxon rank-sum test. Overall, recurrence-free and disease-free survival curves were obtained utilizing Kaplan-Meier. Associations between prognostic factors and survival outcomes were tested on univariate and multivariate Cox models. In multivariate analysis, cox-proportional hazard regression analyses were performed in variables with P values <0.15 on univariate analyses. *P-*value less than 0.05 indicates statistical significance. All statistical analyses were performed on SPSS 26.0 software (IBM, Armonk, NY).

## Results

### Risk Factors Associated With the Recovery of Cord Mobility

A total of 78 patients with HPSCC were included in this study. There were 75 males and 3 females, with a mean age of 57.5 years (range 36–80 years). Before the initial treatment, 56 (71.8%) patients had fixed vocal cords, and 22 (28.2%) patients had impaired vocal cords. Fifty-six (71.8%) patients had improved cord mobility after non-surgical treatment. The rates of SD, PD, PR, and CR were 24.4% (19/78), 3.8% (3/78), 12.8% (10/78), and 59.0% (46/78), respectively. Details of demographic characteristics between the IM and non-IM groups are summarized in **Table 1**. Patients who had no clinical evidence of lymph node metastasis (cN0) (*P=*0.016) and who did not undergo pretreatment tracheotomy (*P <*0.001) were more likely to have IM after non-surgical treatment than patients with clinical evidence of lymph node metastasis and those who underwent pretreatment tracheotomy. Patients with postcricoid region cancers had a tendency of having IM, but the difference did not reach statistical significance (*P=*0.080).

**Table 1:** Demographic characteristics between the improved mobility (IM) and non-IM groups.

		Total n	Improved group n (%)	Non-Improved group n (%)	*P* value
Numbers		78	56 (71.8)	22 (28.2)	
Age (mean ± standard deviation)		57.47 ± 10.266	56.91 ± 10.001	58.91 ± 11.023	0.465
Gender					1.000
	Female	3	2 (3.6)	1 (4.5)	
	Male	75	54 (96.4)	21 (95.5)	
Primary site					0.080
	Pyriform sinus	63	45 (80.4)	18 (81.8)
	Posterior wall	9	5 (8.9)	4 (18.2)
	Postcricoid region	6	6 (10.7)	0
Cord mobility status before treatment					
	Fixed	56	40 (71.4)	16 (72.7)	1.000
	Impaired	22	16 (28.6)	6 (27.3)
	Ipsilateral	75	55 (98.2)	20 (90.9)	0.190
	Bilateral	3	1 (1.8)	2 (9.1)
T classification					0.316
	T2	6	3 (5.4)	3 (13.6)
	T3	29	23 (41.1)	6 (27.3)
	T4	43	30 (53.6)	13 (59.1)
N classification					0.028^*^
	N0	12	12 (21.4)	0
	N1	6	4 (7.1)	2 (9.1)
	N2	53	36 (64.3)	17 (77.3)
	N3	7	4 (7.1)	3 (13.6)
Pretreatment tracheotomy					<0.001^*^
	Yes	12	3 (5.4)	9 (40.9)
	No	66	53 (94.6)	13 (59.1)
Salvage surgery					0.243
	Yes	19	16(28.6)	3(13.6)
	Partial pharyngectomy	5	5 (8.9)	0
	Total pharyngolaryngectomy	7	5 (8.9)	2 (9.1)
	Neck dissection alone	7	6 (10.7)	1 (4.5)
	No	59	40(71.4)	19(86.4)
Systemic therapy					
	Concurrent chemoradiation	39	32 (57.1)	7 (31.8)	0.07
	Neoadjuvant chemotherapy	22	15 (26.8)	7 (31.8)	0.781
	Targeted therapy	22	16 (28.6)	6 (27.3)	1.000

*P < 0.05. All p value below 0.05 were highlighted in bold values.

### Outcomes Between Two Groups

Six patients were lost to follow-up after non-surgical treatment: two in the non-IM group and four in the IM group. Eight patients occurred heterochronic secondary primary cancers after initial treatment, seven patients had esophageal carcinomas and one patient had soft palate carcinoma. Twenty-three patients (44.2%) had recurrence in IM group comparing to 16 patients (76.2%) in non-IM group (**Table 2**). Non-improved cord mobility was significantly associated with recurrence (*P=*0.019), especially local-regional recurrence (*P=*0.001). In patients who did not accept salvage surgery, if non-improved cord mobility was regarded as an indicator for local-regional recurrence within 1 year, the sensitivity, the specificity, positive predictive value, and negative predictive value were 60.9, 87.5, 77.8, 75.7% respectively.

**Table 2:** Outcomes between improved mobility (IM) and non-IM groups.

		IM group	Non-IM group	*P* value
Secondary primary cancer		7	1	0.425
Recurrence		23	16	0.019^*^
	Local-regional recurrence	14	15	0.001^*^
	Distant metastasis	12	4	1.000
Local-regional recurrence within 1 year^#^				<0.001^*^
	Yes	9	14	
	No	28	4	
Overall patients (%)				
	5-year RFS	53.2	21.8	0.002^*^
	5-year DFS	48.0	21.8	0.005^*^
	5-year OS	61.5	30.0	<0.001^*^
With salvage surgery (%)				
	5-year RFS	64.3	33.3	0.301
	5-year DFS	57.1	33.3	0.378
	5-year OS	76.2	66.7	0.611
Without salvage surgery (%)				
	5-year RFS	48.3	20.0	0.005^*^
	5-year DFS	43.9	20.0	0.010^*^
	5-year OS	56.1	23.5	0.001^*^

^#^Patients, who did not accept salvage surgery, occurred local-regional recurrence or not. *P < 0.05; All p value below 0.05 were highlighted in bold values.

OS, overall survival; RFS, recurrence-free survival; DFS, disease-free survival; IM, improved cord mobility; non-IM, non-improved cord mobility.

**Figure 1:**
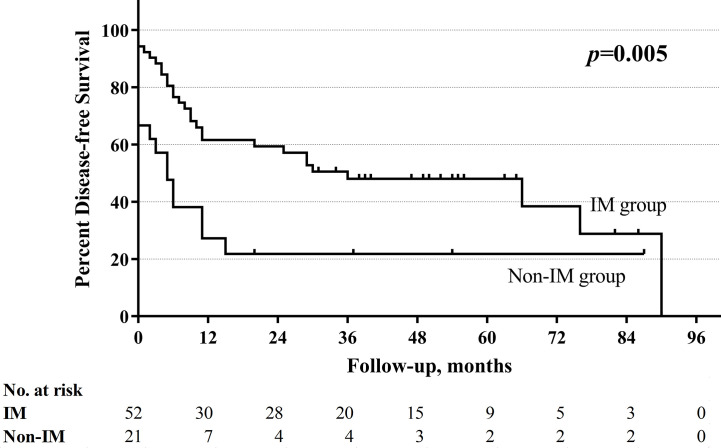
Disease-free survival curve of improved mobility (IM) group and non-IM group.

**Figure 2:**
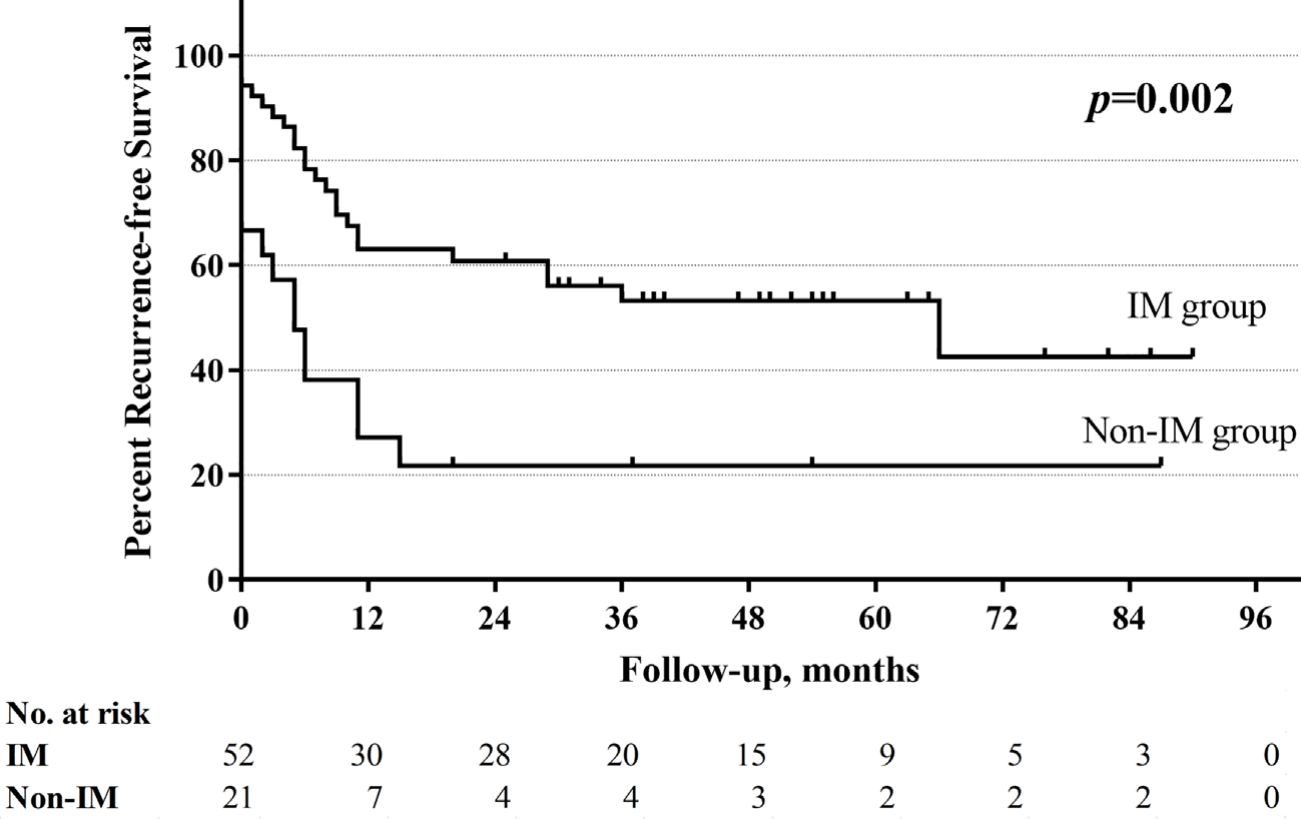
Recurrence-free survival curve of improved mobility (IM) group and non-IM group.

**Figure 3:**
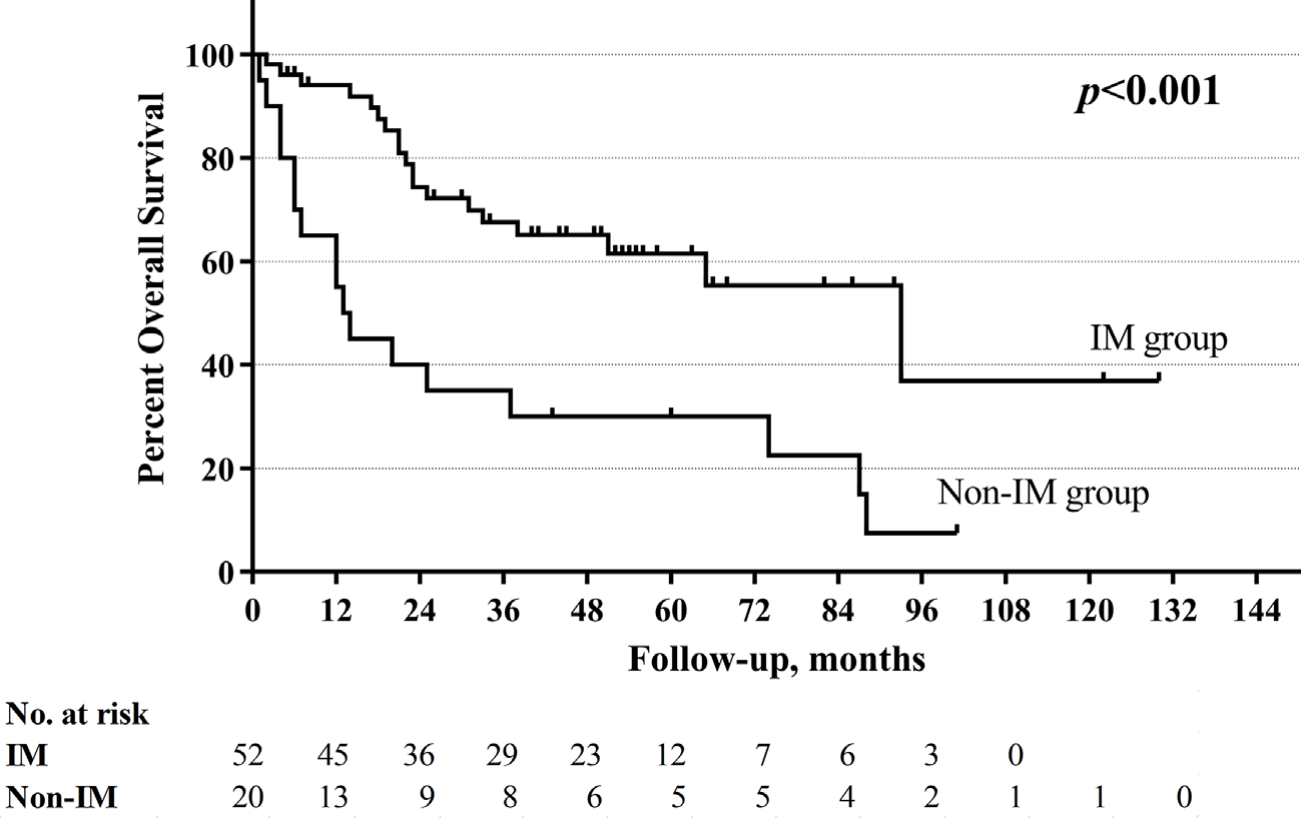
Overall survival curve of improved mobility (IM) group and non-IM group.

With a median follow-up of 33.5 months (range 1–130 months), the total 5-year OS rates were 52.4%, respectively. The non-improved cord mobility was significantly associated with shortened DFS (*P=*0.005), RFS (*P=*0.002), and OS (*P*<0.001) (**Figures 1**–**3**). The 1-year DFS rate of the non-IM group was 27.2%. The 5-year RFS and OS rates of the non-IM group (compared to the IM group) were 21.8% (*vs*. 53.2%) and 30.0% (*vs*. 61.5%), respectively.

### Multivariate Analysis of Recurrence-Free Survival, Disease-Free Survival, and Overall Survival

The univariate analysis revealed that a high N classification (*P=*0.016), without salvage surgery (*P=*0.010), non-improved cord mobility (*P*<0.001), pretreatment tracheotomy (*P=*0.046) were associated with poor OS. Furthermore, the multivariate analysis revealed that all of these variables except for pretreatment tracheotomy were independent risk factors (**Table 3**). The mean OS time was 78 months in the IM group *vs*. 34 months in the non-IM group (HR 2.734, 95% CI=1.340 −5.578). The multivariate analysis also revealed that IM was an independent protective factor for prolonged DFS (*P*=0.043) and RFS (*P*=0.030). The median DFS time was 36 months in the IM group *vs*. 5 months in the non-IM group (HR 1.925, 95% CI=1.020–3.636).

**Table 3:** Univariate and multivariate analysis about disease-free survival (DFS), recurrence-free survival (RFS), and overall survival (OS).

	Univariate analysis			Multivariate analysis					
	DFS	RFS	OS	DFS		RFS		OS	
	*P* value	*P* value	*P* value	HR (95% CI)	*P* value	HR (95% CI)	*P* value	HR (95% CI)	*P* value
N classification	<0.001^*^	<0.001^*^	0.016^*^	1.803 (1.033–3.145)	0.038^*^	2.016 (1.066–3.813)	0.031^*^	2.611 (1.354–5.036)	0.004^*^
Pretreatment tracheotomy	0.362	0.285	0.046^*^	/	/	/	/	/	0.228
Primary site	0.820	0.629	0.712	/	/	/	/	/	/
IM/non-IM	0.005^*^	0.002^*^	<0.001^*^	1.925 (1.020–3.636)	0.043^*^	2.074 (1.075–4.002)	0.030^*^	2.734 (1.340–5.578)	0.006^*^
Salvage surgery	0.225	0.138	0.010^*^	/	/	/	0.262	0.262 (0.089–0.772)	0.015^*^
Concurrent chemoradiation	0.352	0.303	0.677	/	/	/	/	/	**/**

OS, overall survival; RFS, recurrence-free survival; DFS, disease-free survival; IM, improved cord mobility; non-IM, non-improved cord mobility.

HR, hazard ratio; CI, confidence interval; *P < 0.05. All p value below 0.05 were highlighted in bold values.

### Salvage Surgery as a Protective Factor for Overall Survival

The multivariate analysis revealed that patients who underwent salvage surgery had higher OS (HR 0.262, 95% CI=0.089–0.772, *P*=0.015) than patients who did not undergo salvage surgery. IM group had obviously higher 5-year RFS, DFS, and OS not only in overall cohort but also in cohort without salvage surgery (**Table 2**). However, in cohort with salvage surgery, there were no significant difference in all kinds of survival between two groups.

**Figure 4:**
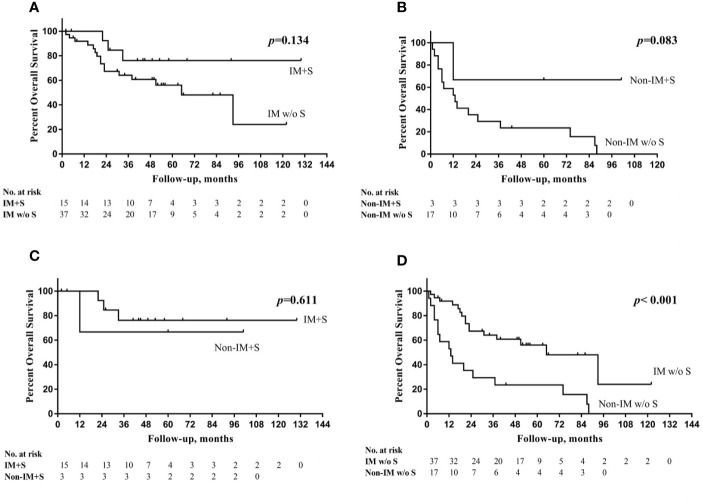
**(A)** Overall survival (OS) curve in IM group with or without salvage surgery (S); **(B)** OS curve in non-IM group with or without S; **(C)** OS curve in IM group with S and non-IM group with S; **(D)** OS curve in IM group without S and non-IM group without S.

Patients were divided into four groups according to the change in cord mobility and the choice of salvage surgery. The mean OS times of these four groups were as follows (from high to low): IM with salvage surgery (105 months), non-IM with salvage surgery (71 months), IM without salvage surgery (67 months), and non-IM without salvage surgery (28 months) (P<0.001). **Figure 4** showed the overall survival curve in four groups.

## Discussion

Although HPSCC is rare (incidence of 5%), it has the worst prognosis of all head and neck squamous cell carcinomas (1, 2, 6). Pretreatment cord dysfunction is regarded as an indicator of an unfavorable prognosis (7, 8). However, the cord mobility status might change after non-surgical treatment. Vocal cord dysfunction might be due to tumor invasion of laryngeal structures, such as the cricoarytenoid joint, thyroarytenoid muscle, and posterior cricoarytenoid muscles, and tumor invasion of recurrent nerves. However, the most common cause of vocal cord immobility can be explained by the weight effect (i.e., the occupancy of the tumor mass). Katilmis et al. (9) found that 50% of vocal cord dysfunction cases were caused by the weight effect because none of the laryngeal structures were involved in these cases based on the histopathological evaluation of total laryngectomy specimens. Therefore, when the tumor mass was eliminated through non-surgical treatment, patients who experienced vocal cord dysfunction caused by the weight effect had a high possibility of experiencing normal mobility. We speculate that the weight effect might partly explain why the rate of improved cord mobility was as high as 71.8% after non-surgical treatment in our study.

Although few studies have evaluated the relationship between changes in the cord mobility status and prognosis in HPSCC, some studies that focused on patients with laryngeal carcinoma and improved cord mobility might provide some insights. Solares et al. (4) analyzed the 5-year local control rate of 23 patients with advanced laryngeal carcinoma who had initial vocal cord fixation and then accepted concurrent chemoradiotherapy. Fifteen patients with improved cord mobility had a 100% 5-year OS rate, whereas those with persistent fixed cord mobility after treatment had a 25% 5-year OS rate (*P*<0.001). However, not all evidence suggests that IM is a predictive factor of good local control. Lee et al. (10) performed a retrospective study on 69 patients with dysfunctional vocal cords and laryngeal cancers. They did not find a significant difference in the 2-year local control rate between patients with IM (53/69) and those without IM (16/69) (70% *vs*. 77%, *P*=0.81). Different from the studies mentioned above, our target population was patients with HPSCC. According to our results, non-improved cord mobility was a strong risk factor for RFS, DFS, and OS. The improved mobility group had a mean OS time of 78 months, which was twice as long as that of the non-IM group. Furthermore, the IM group had a median DFS time seven times longer than that of the non-IM group (36 months *vs*. 5 months). In contrast, non-improved cord mobility might be an indicator for a high possibility of a residual tumor. If non-improved cord mobility was used to predict local-regional recurrence after non-surgical treatment within 1 year, it had a high specificity (87.5%), although the sensitivity was 60.9%. Therefore, we hypothesized that non-improved cord mobility might be a key indicator that should be evaluated to assess the response to non-surgical treatment in addition to imaging examinations, such as contrasted computed tomography (CT), magnetic resonance imaging (MRI), and fluorodeoxyglucose positron emission tomography (FDG-PET). Patients whose cord mobility remained dysfunctional or worsened after non-surgical treatment might need a more aggressive salvage strategy for survival benefits and local control.

The trend in the management of HPSCC is toward a preference for organ preservation without sacrificing survival. However, salvage surgery still has an obvious advantage in the survival of advanced HPSCC patients, especially in those who experience recurrence (11). Al−Mamgani et al. (12) revealed that the total laryngectomy-based strategy resulted in better 5-year local control for T4 laryngeal and hypopharyngeal cancers than organ-preservation chemoradiation (64% *vs*. 87%, *P*=0.030); however, there was no improvement in OS. In the current study, salvage surgery had an independently positive impact on OS in HPSCC patients with pretreatment dysfunctional cord mobility. In the non-IM group, salvage surgery increased the 5-year OS rate from 23.5 to 66.7%. Non-IM group had obviously shorter 5-year RFS, DFS, and OS not only in overall cohort but also in cohort without salvage surgery. However, in cohort with salvage surgery, there were no significant difference between two groups in all kinds of survival outcomes and the 5-year OS of non-IM group became closed to IM group (66.7% *vs*. 76.2%, *P*=0.611). We speculate that patients with non-IM might obtain a survival benefit from salvage surgery, but we cannot draw a conclusion because of the small sample size.

When predicting the prognosis of HPSCC, several risk factors, such as T classification, N classification, pretreatment cord fixation, and pretreatment tracheotomy dependence, should be considered (13). Ho et al. (14) retrospectively analyzed the survival rate of 8,351 patients with hypopharyngeal and laryngeal cancers. Their univariate and multivariable models suggested that metastatic lymph node burden and extranodal extension were two crucial risk factors for mortality. In accordance with the previous study, our results demonstrated that N classification was an independent risk factor for OS, RFS, and DFS. In a multi-institutional study containing 226 patients with advanced laryngeal cancers, 31.4% (71/226) underwent pretreatment tracheotomy. Moreover, their results showed that patients who underwent pretreatment tracheotomy had a statistically significant decrease in both OS (HR 1.55, 95% CI=1.03−2.34, *P*=0.03) and DFS (HR 1.54, 95% CI = 2.07−2.22, *P*=0.02) (15). Some studies have also revealed that pretreatment tracheotomy was a strong predictor for long-term tracheostomy after organ-preservation strategy in laryngeal and hypopharyngeal cancers (16, 17). In our study, which focused on HPSCC patients with pretreatment cord dysfunction, pretreatment tracheotomy was associated with OS only in the univariate analysis, but the difference was not statistically significant in the multivariate analysis. Interestingly, we found that N classification and pretreatment tracheotomy were two pretreatment predictors associated with the possibility of improved cord mobility. Patients with N0 classification and who did not undergo pretreatment tracheotomy were likely to experience improved cord mobility after non-surgical treatment.

This study had certain limitations. First, since our target population was HPSCC patients with pretreatment dysfunctional cord mobility, the results cannot be extensively applied to all patients with HPSCC. Additionally, there were few females in the study. Hypopharyngeal cancers are more common in the male gender. The prevalence of hypopharyngeal cancer in male gender was shown much higher based on our findings, consistent with that in other literatures from Asian (18–20). Our conclusion may be more applicable for the male patients due to the unbalanced gender distribution. Second, due to the small sample size, we could not perform further analyses to ensure that salvage surgery had a certain survival benefit for patients without improved cord mobility. Last but not least, limited by the retrospective nature and the long inclusion time, this study could not avoid bias from the preference of patients and doctors. Moreover, because of the retrospective nature of the study, we were unable to evaluate the functional outcomes. Additional evidence from randomized controlled trials is required to evaluate the prognostic value of salvage surgery for HPSCC patients without improved cord mobility. The further study that focused on the relationship between improved cord mobility and functional outcomes should also be considered.

## Conclusion

The change in cord mobility should be evaluated after non-surgical treatment because non-improved cord mobility is a key indicator to predict poor prognosis. In total, 71.8% of patients with fixed/impaired vocal cords pretreatment improved after non-surgical treatment. Improved cord mobility and salvage surgery both had an independently positive impact on OS in HPSCC patients with pretreatment dysfunctional cord mobility. Non-improved cord mobility may indicate a high possibility of a residual tumor, therefore, patients whose cord mobility remains dysfunctional or worsens after non-surgical treatment might need an aggressive salvage strategy.

## Data Availability Statement

The raw data supporting the conclusions of this article will be made available by the authors, without undue reservation.

## Ethics Statement

The studies involving human participants were reviewed and approved by the institutional review board of the National Cancer Hospital of the Chinese Academy of Medical Sciences. The patients/participants provided their written informed consent to participate in this study.

## Author Contributions

Y-qH: study design, data acquisition, data analysis and interpretation, statistical analysis, manuscript preparation, manuscript editing, manuscript review, approval of final manuscript, agrees to be accountable. X-wZ: data acquisition, data analysis, manuscript editing; manuscript review, approval of final manuscript, agrees to be accountable. Y-mZ: study concept, manuscript editing, manuscript review, approval of final manuscript, agrees to be accountable. X-gN: data acquisition, manuscript editing, manuscript review, approval of final manuscript, agrees to be accountable. Z-hH: data acquisition, manuscript editing, manuscript review, approval of final manuscript, agrees to be accountable. C-mA: data analysis, manuscript editing, manuscript review, approval of final manuscript, agrees to be accountable. J-lY: study concept, data analysis, manuscript editing, manuscript review, approval of final manuscript, agrees to be accountable. S-yL: study concept, study design, manuscript editing, manuscript review, approval of final manuscript, agrees to be accountable. All authors contributed to the article and approved the submitted version.

## Funding

This research was supported by the Non-profit Central Research Institute fund of Chinese Academy of Medical Sciences. Grant/Award Number: Grant No.2019-RC-HL-004.

## Conflict of Interest

The authors declare that the research was conducted in the absence of any commercial or financial relationships that could be construed as a potential conflict of interest.
